# Veterinary Medical Association of New Jersey

**Published:** 1892-10

**Authors:** W. H. Cooper

**Affiliations:** Sec’y.; 47 Montgomery St., Jersey City


### SOCIETY PROCEEDINGS.

*Veterinary Medical Association of New Jersey.*—The Veterinary Medical Association of New Jersey held its 26th regular meeting on Thursday, August 11, 1892, at Sænger Hall, Belmont Ave., Newark, N. J.

The meeting was called to order at 11 A.M. by the president, Dr. J. C. Dunstan, of Morristown, N. J. The Secretary (W. H. Cooper), called the roll and the following responded: Dr. J. W. Hawk, Newark; Dr. W. B. E. Miller, Camden; Dr. Jas. C. Dustan, Morristown; Dr. W. H. Cooper, Jersey City; Dr. J. C. Higgins, New Brunswick; Dr. J. Gerth, Newark; Dr. B. F. King, Little Silver; Dr. M. M. Stage, Dover; Dr. S. L. Lockwook, Woodbridge; Dr. J. M. Everitt, Hackettstown; Dr. H. W. Rowland, Newark; Dr. A. W. Axford, Naughright; Dr. W. Runge, Newark; Dr. H. Exton, Washington; Dr. R. C. Hasbrouck, Passaic; Dr. S. W. Schuppan, Jersey City; Dr. B. L. Drummond, Woodbridge: Dr. H. W. Eliot, Newark, and Dr. J. N. Wittpenn and Dr. O. C. Eisenhart as applicants.

A quorum being present, the chair ordered the minutes of the last meeting read, which, after the reading, were approved. The President made the regular address. The Secretary's report read, was ordered on file. The Treasurer's report made and ordered on file.

Applications for membership: Dr. J. N. Wittpenn, graduate American Veterinary College; Dr. O. C. Eisenhardt, graduate Ont. Vetinary College; Dr. F. Lippincott, non-graduate. Drs. Wittpenn and Eisenhart were elected active members and Dr. Lippincott was returned. Dr. W. Horace Hoskins, Philadelphia, was elected an honorary member of the Association, and John O. Pitney, the Association counsellor of Newark, was also elected an honorary member.

The Board of Censer read their report and it was received. Report of the special meeting of Board of Trustees held in Newark two weeks ago on registration, was read and received. Also report of the prosecution of a case in Camden. Secretary read letters to the Association which were ordered on file.

Dr. S. W. Schuppan read a paper, "Is Pneumonia Contagious?" He said: ' Pneumonia presents many phases as we meet it from day to day, and we are impressed with the variety of symptoms and still more of causes. These causes we find are numerous, and we try to form an opinion upon the individual case in hand; one horse of a team is often only affected when both are worked, handled and exposed alike.

" In pneumonia there is an expectoration which is oftentimes, and I might say generally, swallowed by our patients. A discharge from the nostrils drops onto the ground, dries, becomes pulverized and soon there is a substance which is capable of floating in the air. Now what shall we call this, the contagium?" He gave his experience in two large barns:

" A grey mare sixteen hands high, weighing 1400 pounds, was taken with simple pneumonia, the course of the disease was, as in all recoveries, gradually getting better each day. Six days after I saw her, a bay horse standing next to this developed broncho-pneumonia. These horses belonged to different teams, driven by different men in hauling coal, as all the horses in this barn. Now the question arises, what caused the second case of pneumonia? Can we say exposure or inhalation of irritating gases? The second horse continued to improve when the owner informed the attending surgeon that his horse had had enough medicine, and it died two days later.

"Another barn, branch of a large livery stable, contained six horses. Here we had a well-developed simple pneumonia which was in progress but four days when the second horse showed symptoms of the disease. In the course of the following few days the others all contracted pneumonia, some broncho, some bi-lateral, and one having both lungs and the pleura affected. The sanitary conditions of the small barn were much better than in the larger or main barn. None of this lot of horses died, the recovery taking much longer in the last horses affected.

"He reviewed the present biology which has discovered a microbe called Pneunococcus. If such a thing exists, why is not pneumonia contagious? The microbe, it is something very minute, having power to live, move and propagate, and such a thing we find in the mucous and nasal discharges. If this theory is accepted, why does not the chain of argument give us a contagious disease? To a slight degree, to be sure.

"He recalled the experiments and discovery of Belviny and Kitasato in 1890. That the blood serum taken from animals which had been rendered immune to tetanus and diphtheria could cure other animals so affected, and led the way to the investigations of Drs. Klemperer and Emmerich of causing immunity from pneumonia by inoculation. Dr. Emmerich subjected a rabbit to an intravenous injection of a very dilute, but completely virulent injection of the Diplococco Pneumonial, and rendered it immune. Rabbits so prepared were killed, skinned, divided and rubbed into a fine paste which was pressed through the meshes of a sterilized cloth. Twenty-one rabbits, one of which had been subjected to an injection of the immunizing juice, were subjected in an inhalation apparatus to the action of a spray of bouillon culture of diplococci. All died speedily, except the one animal protected by the immunizing juice.

"Other experiments were tried with the same results, showing almost conclusively the result of the propagation of pneumonia baccilli. Some authors say it takes from three to four days from the time of injection until immunity is produced. Thus this does not aid us in treatment, but in prevention."

Cases were reported by Dr. Miller, Camden; Dr. Gerth, Newark; Dr. Higgins, New Brunswick; Dr. Runge, Newark; Dr. Schuppan, Norwalk, Conn.

The following names were dropped from the roll for non-payment of dues: Dr. Otto Von Lang, Salem; Dr. C. Lawrenz, Newark; Dr. Robert Leis, Newark; Dr. R. T. Churchill, Secaucus; Dr. R. Stanwood, Frelhold; Dr. A. Maurice, Moorstown.

After the committees' reports the meeting adjourned for dinner. The meeting was recalled at 4 P.M., and transacted miscellaneous business.

Adjourned at 7 P.M., to meet on Thursday, Dec. 8, 1892, at New Brunswick, N. J.

W. H. Cooper, *Sec'y.*

47 Montgomery St., Jersey City.

